# Distribution of *Plasmodium* species on the island of Grande Comore on the basis of DNA extracted from rapid diagnostic tests

**DOI:** 10.1051/parasite/2016034

**Published:** 2016-08-26

**Authors:** Nasserdine Papa Mze, Ambroise D. Ahouidi, Cyrille K. Diedhiou, Rahamatou Silai, Mouhamadou Diallo, Daouda Ndiaye, Mbacké Sembene, Souleymane Mboup

**Affiliations:** 1 Laboratory of Bacteriology-Virology, Hospital Aristide le Dantec Dakar BP 7325 Senegal; 2 Laboratory of National Malaria Control Program Moroni Comoros; 3 Laboratory of Parasitology and Mycology, Faculty of Medicine and Pharmacy, Cheikh Anta Diop University BP 5005 Dakar Senegal; 4 Faculty of Science and Technology, Department of Animal Biology, Cheikh Anta Diop University Dakar Senegal

**Keywords:** *Plasmodium* species, RDT, Nested PCR, Malaria, Comoros

## Abstract

In the Union of Comoros, interventions for combating malaria have contributed to a spectacular decrease in the prevalence of the disease. We studied the current distribution of *Plasmodium* species on the island of Grande Comore using nested PCR. The rapid diagnostic tests (RDTs) currently used in the Comoros are able to identify *Plasmodium falciparum* but no other *Plasmodium* species. In this study, we tested 211 RDTs (158 positive and 53 negative). Among the 158 positive RDTs, 22 were positive for HRP2, 3 were positive only for pLDH, and 133 were positive for HRP2 and pLDH. DNA was extracted from a proximal part of the nitrocellulose membrane of RDTs. A total of 159 samples were positive by nested PCR. Of those, 156 (98.11%) were positive for *P. falciparum*, 2 (1.25%) were positive for *P. vivaxI*, and 1 (0.62%) was positive for *P. malariae*. None of the samples were positive for *P. ovale*. Our results show that *P. falciparum* is still the most dominant species on the island of Grande Comore, but *P. vivax* and *P. malariae* are present at a low prevalence.

## Abstract (fr)

Dans l’Union des Comores, des interventions pour la lutte contre le paludisme ont contribué à une diminution spectaculaire de la prévalence de la maladie. Nous avons étudié la répartition actuelle des espèces de *Plasmodium* sur l’île de Grande Comore par PCR imbriquée. Les tests de diagnostic rapide (TDRs) actuellement utilisés aux Comores sont en mesure d’identifier *Plasmodium falciparum*, mais pas d’autres espèces de *Plasmodium*. Dans cette étude, nous avons testé 211 TDRs (158 positifs et 53 négatifs). Parmi les 158 TDRs positifs, 22 étaient positifs pour HRP2, 3 étaient positifs seulement pour pLDH et 133 étaient positifs pour à HRP2 et pLDH. L’ADN a été extrait d’une partie proximale de la membrane de nitrocellulose des TDRs. Au total, 159 échantillons étaient positifs par PCR nichée. Parmi eux, 156 (98,11 %) étaient positifs pour *P. falciparum*, 2 (1,25 %) étaient positifs pour *P. vivax* et 1 (0,62 %) était positif pour *P. malariae*. Aucun des échantillons n’était positif pour *P. ovale*. Nos résultats montrent que *P. falciparum* est toujours l’espèce dominante dans l’île de Grande Comore, mais *P. vivax* et *P. malariae* sont présents à une faible prévalence.

## Introduction

Malaria is a disease caused by a parasite that belongs to the genus *Plasmodium*. It is responsible for about 627,000 deaths worldwide annually with about 90% occurring in sub-Saharan Africa [19]. In the Union of Comoros (consisting of three islands: Grande Comore, Moheli and Anjouan), located in the Indian Ocean between Madagascar and the African coast, malaria is one of the major public health concerns, with *Plasmodium falciparum* being the most prevalent species in this region [17]. Therefore, three major strategies were implemented in 2004, comprising the use of insecticide-treated bed nets, introduction of Intermittent Preventive Treatment (IPT) in pregnant women, and the treatment of patients with artemisinin-based combination therapy. These strategies have resulted in a significant decrease in malaria transmission. The prevalence of malaria among pregnant women decreased from 30.4% to 8% between 2004 and 2008, and the hospital case fatality rate among children under 5 years dropped from 0.36% to 0.12% between 2005 and 2008 (PNLP 2004–2009, unpublished data). This decline allowed the National Malaria Control Program to bring into focus strategies for malaria eradication in the Union of Comoros, which was introduced for the first time in 2007 on Moheli Island. The population of the island was given mass drug administration using Artequick^®^ (artemisinin-piperaquine) and primaquine. The program was later introduced in 2012 on the island of Anjouan and in late 2013 on Grande Comore. The results are encouraging: no deaths were recorded in the first quarter of 2014 (PNLP 2014, unpublished data).

The significant decrease of malaria may also induce a change in the distribution of *Plasmodium* species in the Union of Comoros. For better treatment and intervention for malaria elimination, it is necessary to know the current distribution of *Plasmodium* species. This will also enable us to assess the existing approach against malaria in the Union of Comoros.

A study on the distribution of *Plasmodium* species by microscopy conducted in the Union of Comoros showed that *P. falciparum* is the most prevalent species [17]. Microscopy analysis remains the standard method for the diagnosis of malaria. However, it requires expertise particularly when the parasitemia is low but also in case of mixed infections [20]. In developing countries, microscopists are not sufficiently trained and this can lead to misidentification of the *Plasmodium* species [9]. The use of PCR methods can help to fill this gap.

The study’s goal was to reassess the distribution of the *Plasmodium* species using rapid diagnostic tests (RDTs) DNA by PCR, in areas where malaria is hypoendemic (Moroni), mesoendemic (Mitsamiouli), and meso to hyperendemic (Mbeni) [16] (PNLP 2004–2009, unpublished data).

## Materials and methods

### Study population

Positive and negative rapid diagnostic tests (RDTs) performed in febrile patients (Malaria pLDH/HRP2 Combo: Access Bio, PBX-KM30003 and SD Bioline Malaria Ag Pf/Pan: SD Bioline, 05FK60) for malaria diagnosis were collected in Grande Comore, at the National Malaria Control Program in the capital city Moroni and at two hospitals of two regions of Grande Comore: Mitsamiouli and Mbeni from 2012 to 2013. These RDTs detect the presence of both the HRP2 protein, specific to *P. falciparum*, and the pLDH protein, common to *P. falciparum* and the other three species: *Plasmodium malariae*, *Plasmodium vivax*, and *Plasmodium ovale*. Only the RDTs with a valid control band were included in this study.

Two hundred eleven RDTs were used, including 158 positive RDTs (14 for the kit SD Bioline Malaria Ag Pf/Pan Malaria and 144 pLDH/HRP2 Combo kit), and 53 negative RDTs (all done with the Combo Kit). Among the 158 positive samples, 22 were positive only for HRP2, 133 were positive for HRP2 and pLDH, and 3 were positive only for pLDH. Among the positive ones, 29 came from Moroni, 52 from Mitsamiouli, and 77 from Mbeni.

### DNA extraction

Parasite DNA was extracted from RDTs using a proximal part of the nitrocellulose membrane (1/3 NC) as previously described by Cnops et al. [6]. DNA was extracted with the QIAamp DNA Mini kit (Qiagen) according to the manufacturer’s recommendations for filter paper.

### DNA amplification

Species-specific *Plasmodium* identification nested PCR was performed using the following primers [18]: 5′-CCT GTT GTT GCC TTA AAC TTC-3′, and 5′-TTA AAA TTG TTG CAG TTA AAA-3′. PCR was carried out using the following conditions: Initial 4-min denaturation at 94 °C followed by 35 cycles with 30-s at 94 °C, 1-min at 55 °C, and 4-min final extension at 72 °C. 1 μL of the nest-1 amplification products was used as DNA template for the nest-2 amplification.

The following primers specific to each species were used for the nest-2 PCR: 5′-TTA AAC TGG TTT GGG AAA ACC AAA TAT ATT-3′, 5′-ACA CAA TGA ACT CAA TCA TGA CTA CCC GTC-3′ for *P. falciparum*, 5′-CGC TTC TAG CTT AAT CCA CAT AAC TGA TAG-3′, 5′-ACT TCC AAG CCG AAG CAA AGA AAG TCC TTA-3′ for *P. vivax*, 5′-ATA ACA TAG TTG TAC GTT AAG AAT AAC CGC-3′, 5′-AAA ATT CCC ATG CAT AAA AAA TTA TAC AAA-3′ for *P. malariae*, and 5′-GGA AAA GGA CAC ATT AAT TGT ATC CTA GTG-3′, 5′-ATC TCT TTT GCT ATT TTT TAG TAT TGG AGA-3′ for *P. ovale*. PCR was carried out using the following conditions: Initial 4-min denaturation at 94 °C followed by 35 cycles with 30-s at 94 °C, 1-min at 58 °C, and 4-min final extension at 72 °C.

PCR products were analyzed on 2% agarose gel. The size of DNA bands obtained was analyzed by GeneRuler 100 bp DNA ladder marker (Quick Load^®^, 100pb Ladder DNA).

### Statistical analysis

The data were analyzed by EpiTools software (Z-test to compare sample proportion). The *p*-value was determined and the threshold of significance was estimated at 0.05 for all statistical tests.

## Results

In this study, 211 RDTs (158 positive and 53 negative) were successfully tested by nested PCR. Among the 158 positive RDTs, 2 (1.3%) were found negative by PCR; and among the 53 negative RDTs, 3 (5.7%) showed positive results for *P. falciparum* by PCR.

Among the 159 positive RDTs by nested PCR, 156 were positive for *P. falciparum* (98.11%), 2 for *P. vivax* (1.25%), and 1 for *P. malariae* (0.62%). All samples were negative for *P. ovale*. No mixed infections were identified. Among 22 RDTs that presented a positive result for HRP2, 20 were positive for *P. falciparum* by PCR and 2 were negative for all species. On the other hand, 133 RDTs that had a positive band for *P. falciparum* (HRP2 positive) or both *P. falciparum* and non-*P*. *falciparum* (HRP2 and pLDH positive) were *P. falciparum* positive by PCR. Among the 3 positive RDTs for the detection of pLDH, two were positive by PCR for *P. vivax* and one for *P. malariae*.

In the capital Moroni, 25 samples were positive for *P. falciparum*, 1 sample was positive for *P. vivax*, and 1 sample was positive for *P. malariae*. In Mbeni, 76 samples were positive for *P. falciparum* and 1 sample was positive for *P. vivax*. In Mitsamiouli, all species were positive for *P. falciparum* (Table 1).

**Table 1.** The distribution of *Plasmodium* species in the three regions of the island of Grande Comore

Sites	Moroni	Mitsamiouli	Mbeni	
	Endemicity			
	Hypoendemic	Mesoendemic	Meso to hyperendemic	Total
	*n* = 27	*n* = 55	*n* = 77	
*P. falciparum*	25 (92.6%)	55 (100%)	76 (98.7%)	156
*P. vivax*	1 (3.7%)	0	1 (1.3%)	2
*P. malariae*	1 (3.7%)	0	0	1
*P. ovale*	0	0	0	0

We investigated whether there was an association between the distribution of species and the endemicity using the Z-test. A significant difference was found for the prevalence of *P. falciparum* between Moroni and Mitsamiouli (*p* = 0.041) and between Moroni and Mbeni (*p* = 0.0345), whereas no difference was observed between Mitsamiouli and Mbeni (*p* = 0.84). Concerning the prevalence of *P. vivax*, a significant difference was found between Moroni and Mbeni (*p* < 0.0001).

## Discussion

To contribute to malaria elimination in the Comoros, we evaluated the current distribution of *Plasmodium* species using DNA extracted from RDTs. In this study, nested PCR was used to determine the *Plasmodium* species, because current RDTs used in the Comoros can specify only *P. falciparum*; microscopy requires more expertise and can be less sensitive [7].

Two RDTs that were found to be positive for *P. falciparum* were negative by PCR. These false positive results could be due mostly to the persistence of HRP2 in recently cured *P. falciparum* infected patients [1, 14], but also to the presence of auto-antibodies [10] or anti-rheumatic factors [3, 12].

Three samples were negative by RDTs, but were positive for *P. falciparum* by PCR. These false negative results could be due to low parasitemia [13] or HRP2 deletion in some isolates of *P. falciparum* [11]. Another explanation could be the presence of anti-antibodies in serum to HRP2 [5], or the presence of an inhibitor in the blood, preventing the occurrence of the *P. falciparum* control band [8]. Therefore, in these three cases only the HRP2 band should be present. The false negative results for *P. falciparum* found in this study were also found in previous studies using the Malaria pLDH/HRP2 Combo test [4, 21]. However, we did not obtain false negatives for species other than *P. falciparum*, unlike previous studies [4, 21], perhaps because the negative RDTs used in our study were moderate in number.

It is therefore important to better monitor false positives and false negatives for better calculation of the prevalence of malaria. Misidentification of the *Plasmodium* agent can delay malaria treatment and may also lead to complications or increase the risk of antimalarial resistance [2].

A total of 133 RDTs had a positive band for *P. falciparum* and a positive band for *Plasmodium spp.* This result suggests that these samples could be positive for only *P. falciparum*, or they could be mixed *Plasmodium* species infections (*P. falciparum* plus other species). However after PCR, we found that all RDTs were positive only for *P. falciparum*. This result confirms that the presence of mixed species is not common in the Comoros [15, 17].

We also determined the *Plasmodium* species distribution by PCR and have shown a prevalence of 98.11%, 1.3%, and 0.6%, respectively, for *P. falciparum*, *P. vivax*, and *P. malariae.* However, Ouledi in 1995 found prevalence rates of 90% for *P. falciparum*, 8% for *P. malariae*, and 1.5% for *P. vivax* [15]. A significant decrease of the prevalence of *P. malariae* (*p* = 0.010) and a significant increase of the prevalence of *P. falciparum* (*p* = 0.0172) have been found between our results and the results reported by Ouledi. A study conducted in 2011 [17] found a prevalence of 96% for *P. falciparum*, 2% for *P. malaria*, and 1.5% for *P. vivax*. For this study, no significant difference was observed for the prevalence of *P. falciparum* (*p* = 0.3802), *P. vivax* (*p* = 0.879), and *P. malariae* (*p* = 0.309).

We found that the prevalence of *P. falciparum* in Mitsamiouli and Mbeni was higher than in Moroni. This difference can be explained by the fact that Mitsamiouli and Mbeni are areas where malaria is mesoendemic and meso to hyperendemic, respectively, while in Moroni malaria is hypoendemic.

*P. ovale* was not found in this study, whereas in the previous results it was observed at a prevalence of 0.5% [15, 17]. The absence of *P. ovale* species may be explained by the fact that in this study, we collected samples only from Grande Comore, whereas the two previous studies were carried out on the three islands of the Union of Comoros. The presence of non-*P*. *falciparum* species in our study and previous studies is important information to use for malaria diagnosis on the island. These results show that it is important to regularly monitor the prevalence of the different *Plasmodium* species using more sensitive tools such as PCR in the Comoros Islands.

## Conclusion

This study, which determined the prevalence of *Plasmodium* species using PCR performed from DNA extracted from RDTs, confirms the strong predominance of *P. falciparum* in the Comoros. The use of more sensitive tools such as PCR, with a large number of samples for the detection of *Plasmodium* species on the Comoros Islands, will provide more information for malaria elimination on the islands.

## Conflict of interest

The authors declare no conflict of interest in relation with this paper.
